# Chemical Management of *Senecio madagascariensis* (Fireweed)

**DOI:** 10.3390/plants12061332

**Published:** 2023-03-15

**Authors:** Kusinara Wijayabandara, Shane Campbell, Joseph Vitelli, Sundaravelpandian Kalaipandian, Steve Adkins

**Affiliations:** 1 School of Agriculture and Food Science, University of Queensland, Gatton Campus, 4343, QLD, Australia; 2 Department of Agriculture and Fisheries, Brisbane 4102, QLD, Australia

**Keywords:** herbicides, plant density, seed bank, fireweed, management

## Abstract

Fireweed (*Senecio madagascariensis* Poir.) is a herbaceous weed-producing pyrrolizidine alkaloid that is poisonous to livestock. To investigate the efficacy of chemical management on fireweed and its soil seed bank density, a field experiment was conducted in Beechmont, Queensland, in 2018 within a pasture community. A total of four herbicides (bromoxynil, fluroxypyr/aminopyralid, metsulfuron-methyl and triclopyr/picloram/aminopyralid) were applied either singularly or repeated after 3 months to a mix-aged population of fireweed. The initial fireweed plant density at the field site was high (10 to 18 plants m^−2^). However, after the first herbicide application, the fireweed plant density declined significantly (to ca. 0 to 4 plants m^−2^), with further reductions following the second treatment. Prior to herbicide application, fireweed seeds in both the upper (0 to 2 cm) and lower (2 to 10 cm) soil seed bank layers averaged 8804 and 3593 seeds m^−2^, respectively. Post-herbicide application, the seed density was significantly reduced in both the upper (970 seeds m^−2^) and lower (689 seeds m^−2^) seed bank layers. Based on the prevailing environmental conditions and nil grazing strategy of the current study, a single application of either fluroxypyr/aminopyralid, metsulfuron-methyl or triclopyr/picloram/aminopyralid would be sufficient to achieve effective control, whilst a second follow-up application is required with bromoxynil.

## 1. Introduction

Madagascar ragwort (*Senecio madagascariensis* Poir.), commonly known as fireweed in Australia, is a short-lived perennial herb that belongs to the Asteraceae family. It is native to southern Africa and has been introduced to several countries, including Argentina, Brazil, Colombia, Uruguay, Japan, Hawaii, and Australia [1]. Fireweed plants produce pyrrolizidine alkaloids (PAs) that are poisonous to livestock, particularly cattle (*Bos taurus* L.) and horses (*Equus ferus caballus* L.). Several general management approaches, including cultural, physical, chemical, biological, or a combination of these, have been used to manage fireweed. It is known to be susceptible to the action of several selective herbicides [2], spanning several ‘mode of action’ groups [3]. For example, fluroxypyr/aminopyralid (HotShot™, Corteva Agriscience Australia, 67 Albert Avenue, Chatswood, New South Wales, Australia, 2067) and triclopyr/picloram/aminopyralid (Grazon™ Extra, Corteva Agriscience Australia, 67 Albert Avenue, Chatswood, New South Wales, Australia, 2067) are combinations of synthetic auxin herbicides (Group 4) [4]. These disrupt plant cell growth in the newly forming stems and leaves and negatively affect protein synthesis and normal cell division, leading to malformed growth and tissue tumours [4]. Bromoxynil (Bromicide^®^ 200, Nufarm Australia, 103–105 Pipe Road, Laverton North, Victoria, Australia, 3026) acts as a Photosystem II photosynthetic inhibitor (Group 5) while metsulfuron-methyl (Brush-Off^®^, Bayer CropScience Australia, 391–393 Tooronga Road, Hawthorn East, Victoria, Australia, 3123)—a member of the sulfonylurea group of herbicides (Group 2)—impedes the normal function of acetolactate synthase (ALS), a key enzyme in the pathway of biosynthesis of the branched-chain amino acids isoleucine, leucine, and valine [4]. A commonplace recommendation for herbicide control of fireweed in Australia is that plants should be sprayed during the early flowering stage of growth with a follow-up treatment often essential 6 months later [2,5]. According to Sindel and Coleman [2], such herbicide applications are best applied to control fireweed populations during April (Autumn) in Australia.

In terms of herbicide efficacy, 2,4-dichlorophenoxyacetic acid (2,4-D) formulations [5,6] dicamba, glyphosate, MCPA, tebuthiuron, triclopyr [6] bromoxynil, fluroxypyr/aminopyralid, triclopyr/picloram/aminopyralid [2,7], and metsulfuron-methyl [7] have all been found to be active in one or more fireweed growth stages (i.e., seedlings, juvenile or mature plants). However, which herbicide and at what rate they can be legally applied may vary between countries and even between jurisdictions within countries (Pest Plants and Animals Act 2005 and Biosecurity Act 2014).

In Australia, 2,4-D amine (3.2 kg ha^−1^) and 2,4-D sodium salt (2 to 4 kg ha^−1^) have been reported to give good fireweed control without harming the proximate pasture species, such as blue couch (*Digitaria didactyla* Wild), blady grass (*Imperata cylindrica* (L.) Beauv) and white clover (*Trifolium repens* L.) [8]. Similarly, Motooka et al. [6] suggested that in Hawaiian pastures where forage legumes are mixed with grasses, the amine salt formulation of 2,4-D is preferable because of its mild impact on legumes. In contrast, metsulfuron-methyl at 40 to 80 g ha^−1^ provided effective control of fireweed in an Australian study, but it severely damaged legumes (such as *T. repens*) present within the treated pasture [9].

The seed bank is defined as a collection of viable, non-germinated seeds [9] and is an important component of grazed pastures. To develop a suitable, long-term chemical management strategy for any grazed pasture, it is important to have information on the dynamics of the weed and pasture species’ seed banks [10]. The soil seed bank significantly contributes to the regeneration ability and future composition of that pasture community [11]. During a typical Australian Autumn (March–May), with average rainfall (of 94.3 mm in 2019; [12]), most fireweed seeds will germinate in the first 3 months after dispersal from the parent plant, and only a small percentage will remain viable and ungerminated in the seed bank after a year [2]. However, in a relatively drier season, a greater proportion of the seed produced will enter the seed bank and is predicted to maintain its viability there for up to 10 years [2]. In one study, freshly collected fireweed seeds buried 3 cm deep in the soil only lost a small percentage of their viability (from 63 to 54%) in the following 15 months [13]. When Radford [13] assessed the size of the fireweed soil seed bank, they found over 12,000 seeds m^−2^ at a heavily infested site, with most seeds found below 1 cm of depth in the soil profile.

Rapidly reducing or eradicating weed seed banks should be relatively easy if seed production and their placement into the seed bank can be prevented [14]. In addition, determining the seed bank size and structure of grazed pastures will be helpful in determining an effective chemical management approach [14]. To eradicate an invasive weed species like fireweed, it is necessary to not only kill the emerging plants but also deplete the seed bank. Even with high levels of plant mortality, the soil seed bank may still allow populations to reappear in future years [15]. In the estimation of the impact of any chemical management approach in an agroecosystem, knowledge of the germination behaviour of the weed species and its seed bank ecology will be important [16]. By using ecological population indices, such as the Shannon–Weiner index [10], determining the effect of chemical management can be made on the species diversity within treated communities.

Thus, the objectives of this study were to: (1) evaluate the impact of several herbicides, that have all been previously found to be efficacious on fireweed but have different modes of action, on fireweed density, (2) compare a single dose to a repeated dose, and (3) determine their ability to rapidly deplete the fireweed seed bank while maintaining the pasture community species diversity.

## 2. Results

### 2.1. Density of Fireweed Plants following Various Chemical Control Approaches

**Figure 1:**
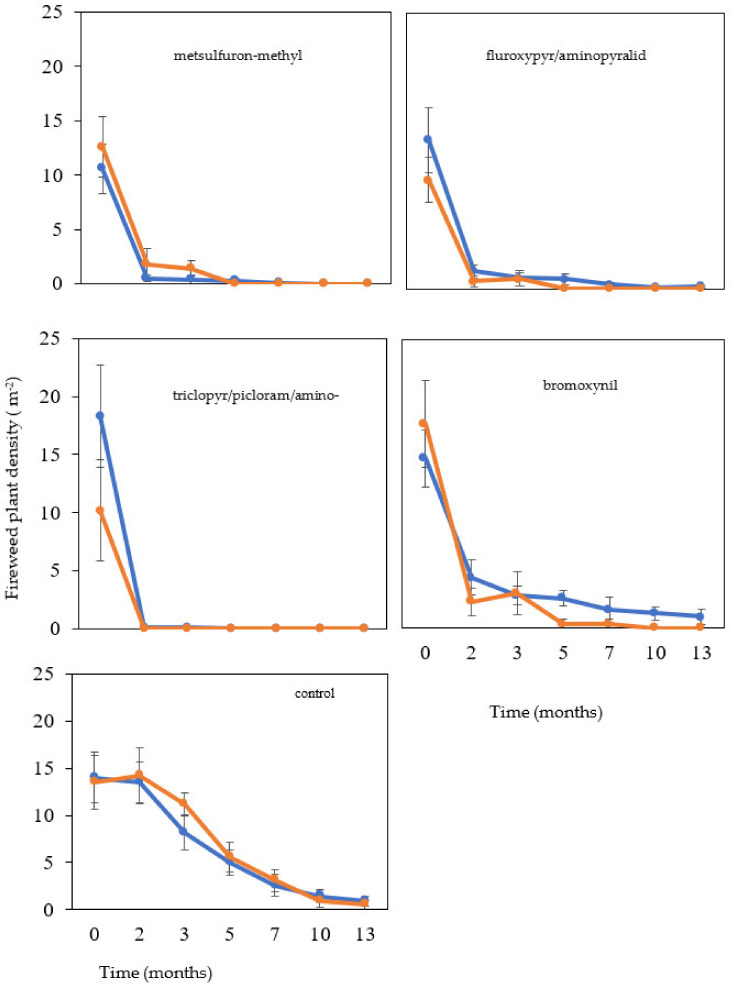
Fireweed plant density (m^−2^) in two different types of field plots: those plots that had been sprayed once (blue line) and those that had been sprayed twice (orange line) recorded over time following the application of metsulfuron-methyl, fluroxypyr/aminopyralid, triclopyr/picloram/aminopyralid, bromoxynil, and control. The data are the mean ± SEM from five replicate plots.

Before applying herbicide treatments, fireweed plant density was relatively high (*ca.* 10 to 18 plants m^−2^) and not significantly different (*p* = 0.56) between treatment plots (Figure 1). Following the first herbicide application, new seedling recruitment was detected in the bromoxynil-treated sub-plots but not in any of the other herbicide treatments. Two months after the first herbicide application, a significant reduction (*p* < 0.05) in fireweed plant density (*ca.* 0 to 4 plants m^−2^) was observed in all herbicide-treated sub-plots when compared to the control sub-plots (*ca.* 14 plants m^−2^; Figure 1).

When comparing the single application plots with the follow-up application plots, the single application had been efficacious for all herbicides except bromoxynil (Figure 1). For bromoxynil, the follow-up application treatment was required to reduce the density of fireweed to zero plants m^−2^, while the single application treatment averaged three plants m^−2^ (Figure 1).

Ten months after application, there was no significant difference (*p* = 0.10) between the single and the follow-up treatments on fireweed density (Figure 1). In addition, during this time, fireweed density had declined in all plots, including the control. At the end of the experiment (i.e., after 13 months), there was a significant difference (*p* < 0.05) in fireweed density in the herbicide-applied plots as compared to the control; however, there was not a significant difference (*p* = 0.15) between the number of applications (apart from the bromoxynil treated plots) (Figure 1).

For the single application sub-plots for metsulfuron-methyl, fluroxypyr/aminopyralid and triclopyr/picloram/aminopyralid applications significantly reduced the density of fireweed compared to the control sub-plots, while no significant difference was observed between bromoxynil and the control (Figure 1). However, following the second herbicide application, the fireweed plant density dropped to 0 in all herbicide-applied sub-plots (including the bromoxynil sub-plots) at the end of the experiment (13 months; Figure 1).

### 2.2. Effect of Chemical Control on Seed Bank Structure

Before the initial application of the herbicides, fireweed seeds were found in both the upper (0 to 2 cm) and lower (2 to 10 cm) soil layers. The fireweed seed density in the upper layer (8804 seeds m^−2^) was significantly (*p* < 0.05) greater than the seed density in the lower layer (3593 seeds m^−2^) (Table 1). The germinable fireweed seed density across the site varied from 7711 to 10,736 germinable seeds m^−2^ in the upper layer and from 3198 to 3972 m^−2^ in the lower layer (Table 1). Fireweed was the most abundant species in the seed bank and accounted for 90% of the total seed bank in both soil layers (Table 1).

There were about 15 species recorded from the pre-treatment seed bank (Table 1). Fireweed, followed by kikuyu (*Pennisetum clandestinum* Hochst. ex Chiov.), had the highest seed density (ranged from 1314 to 4034 seed m^−2^), which is consistent with the study site being a kikuyu-sown pasture. The total seed density of all species, including fireweed in both layers, varied from 18,591 to 26,698 seeds m^−2^. The Shannon–Weiner index for pre-treatment at the top layer (from −0.47 to 1.37) was less than the bottom layer (−0.29 to 1.40) (Table 2).

Following two rounds of herbicide application, the germinable seed bank of fireweed was significantly (*p* < 0.05) reduced (Table 3) due to the absence of any remaining reproductively active plants. Before application, fireweed seeds were found in both upper and lower soil layers, with a significantly (*p* < 0.05) greater portion (7710 to 10,736) in the upper layer (Table 1). When assessed 5 months after the second herbicide application, the germinable fireweed seeds in the upper layer varied from only 601 (fluroxypyr/aminopyralid) to 1263 m^−2^ (Control), while in the lower layer, germinable fireweed seeds varied from 519 (fluroxypyr/aminopyralid) to 866 m^−2^ (bromoxynil) (Table 3). Not only in herbicide-treated sub-plots but also in the control sub-plots, fireweed seed density had dropped by 28%, indicating that loss in seed viability was occurring. Kikuyu was the dominant species in the seed bank, with only 11 other species observed in the seed bank post-herbicide application (Table 3). In addition, nutgrass (*Cyperus rotundus* L.) had increased following herbicide application except in the triclopyr/picloram/aminopyralid treated sub-plots (Table 3). New plant species also appeared (little hogweed, *Portulaca oleracea* L.; burclover, *Medicago polymorpha* L.; Carolina bristle mallow, *Modiola caroliniana* L. G. Don), possibly due to seed dispersal into the site. Whilst some species—which had been present in the first seed bank assessment—were lost from the second seed bank assessment (e.g., white clover, *Trifolium repens* L.; marsh parsley, *Cyclospermum leptophyllum* (Pers.) Sprague ex Britton and P.Wilson; black nightshade, *Solanum nigrum* L.; vervain, *Verbena* spp.; pennywort, *Hydrocotyle* sp. and American pokeweed, *Phytolacca americana* L.), possibly due to short term seed longevity in the seed bank.

The total seed density of the seed bank varied from 4562 to 10,491 seeds m^−2^ (Table 3).

### 2.3. Effect of Chemical Control on Seed Bank Vertical Distribution

There was a significant difference (*p* < 0.05) between the density of the germinable seed banks before and after herbicide application and between the two depths of the soil samples (0 to 2 and 2 to 10 cm), and interaction of these two factors (herbicide application with soil depth) on fireweed seed density in the seed bank. However, there was no significant difference (*p* = 0.12) between the herbicide treatments and the interaction of seed banks with soil depth or with treatment (*p* = 0.67) (Table 4). The Shannon–Weiner Index for post-treatment in the top layer (from 0.85 to 1.64) was higher than in the bottom layer (−0.59 to 1.17) (Table 2).

There was no significant difference in the fireweed seed density within each of the upper layers and lower layers prior to herbicide application in all sub-plots, but the seed density was higher in the upper layer (0 to 2 cm) when compared to the lower layer (2 to 10 cm). However, there was a significant reduction of fireweed density after herbicide application in every treatment as well as in the control sub-plots (Table 4). As compared to the control, the highest reduction of fireweed seed numbers (−18%) was observed in the upper layer of the bromoxynil-treated plots (Table 4).

## 3. Discussion

In the present study, single applications of all the tested herbicides with different modes of action were successful in controlling fireweed growth, except bromoxynil which required a second follow-up application. The bromoxynil treatment was not as effective in a pasture setting, as it is a contact PSII inhibitor herbicide, whereas the other herbicides used were translocatable and thus more effective in a dense grass community. Bromoxynil is most active on smaller fireweed plants, whereas the efficacy of the other translocatable herbicide treatments is less limited by plant size and age. Additionally, bromoxynil seems to be active on a wider range of species, resulting in reduced pasture diversity.

Initially, the fireweed plant density at the Beechmont field site was relatively high (10 to 18 plants m^−2^). However, following the implementation of treatments, all four herbicides rapidly reduced the density of fireweed. The density remained low thereafter, except for the bromoxynil treatment, which showed some evidence of plant regrowth and seedling recruitment. Consequently, implementing a follow-up application of bromoxynil was advantageous, reducing the fireweed density to zero plants m^−2^, while the single application treatments contained three plants m^−2^, 5 months after the first application of bromoxynil.

Watson et al. [5] suggested that a follow-up herbicide treatment was often necessary for effective fireweed management. Similarly, Sindel and Coleman [2] recommended the follow-up of an initial herbicide treatment with spot spraying with one of the registered herbicides, such as triclopyr/picloram/aminopyralid or fluroxypyr/aminopyralid in the Spring. The results from the current study suggest that a second application is not necessary, but if applied, the timing between the two treatments could vary greatly depending on which herbicide is used. For example, follow-up control using bromoxynil may need to be undertaken much sooner than fluroxypyr/aminopyralid, metsulfuron-methyl, or triclopyr/picloram/ aminopyralid (Figure 1). These or similar herbicides have been shown to give some residual control of seedling recruitment for other herbaceous Asteraceae weeds such as florestina (*Florestina tripteris* auth.) [17]. In future, we recommend performing herbicide residual studies on the pasture feed to determine the active substances present in the feed.

Since all the tested herbicides are selective in their mode of control, they are considered not to damage the pasture grass species in the field. However, other components of the pasture community, including the broadleaved pasture legumes, may be damaged (Table 3). This aspect of fireweed management with selective herbicides needs to be further studied. According to Anderson and Panetta [8], clover (*Trifolium* sp.) was severely damaged by metsulfuron-methyl, clopyralid, triclopyr and triclopyr and picloram. Although the 2,4-D formulations damaged neither blue couch (*Digitaria didactyla* Willd.) nor clover, atrazine plus 2,4-D caused severe damage to both species.

In addition, the four tested herbicides have different withholding periods, which may also influence which herbicide is selected for use. Bromoxynil has an 8-week withholding period [2]; therefore, grazing or cutting for stock feed should be avoided during that period (APVMA, 2022). Triclopyr/ picloram/aminopyralid [18] and fluroxypyr/aminopyralid [19] have a 7-day withholding period, and even after death, many plants endure toxicity and more palatable thus, the stock should not be grazing for 7 days [20,21]. However, metsulfuron-methyl has no withholding period [22].

According to the first seed bank analysis, fireweed seeds can be present in the soil in moderately large numbers (3000 to 10,000; Table 2). Ragweed parthenium (*Parthenium hysterophorus* L.), another invasive Asteraceae weed, can form seed banks as large as ca. 45,000 seeds m ^−2^ in a similar habitat to that studied in Southeast Queensland (SEQ) [11]. The results for the fireweed seed bank in this study were like that of Sindel et al. [23], who had undertaken studies in two different locations in New South Wales. A recent study undertaken by Karem [24] has indicated that fireweed seeds collected from the same Beechmont site in SEQ had an indicative short life of <1 year in the seed bank. Since a single fireweed plant can produce up to ca. 30,000 seeds [25] that can be effectively dispersed by wind, the invasive strategy of this weed must be seen as a balance between high seed production with rapid dispersal and producing a medium-sized seed bank of short-lived seeds. Land managers should therefore focus more on preventing seed set and dispersal and, to a lesser extent, on preventing the formation of seed banks. The present study has shown that most of the fireweed seed is to be found in the upper soil layers (0 to 2 cm), while a significant decrease occurs in both layers over time after herbicide application. In the present trial, undertaken in the absence of grazing livestock, this could be due to the use of management practices that do not disturb the soil surface. The field trial site used in this study was not grazed for ca. 10 months following the herbicide treatments, with no cattle to disturb the soil surface, the movement of fireweed seeds deeper into the seed bank of a healthy kikuyu pasture was negligible (Table 3).

According to the Shannon-Weiner index, there was no reduction in the seed community biodiversity in the topsoil layers after herbicide application; however, there was an Index reduction in the bottom layers of bromoxynil-treated sub-plots and in the control sub-plots. This indicates that except for bromoxynil, all other herbicides did not reduce the biodiversity of the pasture seed community. Bromoxynil, being a contact herbicide and effective on seedlings [8], may have reduced biodiversity more than other herbicides. Being a contact herbicide, only those parts of the plant that come directly in contact with the herbicide are killed, and the plant will often regrow from the unaffected parts. Significant fireweed seedling recruitment after spraying is often observed [7]. Anderson and Panetta [8] reported that bromoxynil (3 L ha ^1^) was unsuccessful in controlling mature fireweed plants, with substantial regrowth occurring five months after spraying.

Through natural seed decline and increased competition from the kikuyu pasture, a rapid decline in the soil seed bank is expected to follow. The key to this rapid decline will be the elimination of reproductive plants, a critical component of an effective management strategy for fireweed. Compared to the control, the reduction in seed densities of fireweed in the herbicide-treated sub-plots was high (Table 3). Among tested herbicides, the highest seed density reduction percentage was observed in bromoxynil-treated sub-plots (−18%) (Table 3). Kikuyu was the dominant seed bank species (Table 1) before spraying the herbicides, and there was a dramatic increase of kikuyu seed in the soil seed bank after the herbicides had been applied, indicating the herbicides did not affect kikuyu seed populations or any other grasses or sedges (Table 3). Interestingly, even after the application of herbicide, some new species (*P. oleraceae, M. polymorpha* and *M. carolinia*) appeared from the seed bank while some species declined (*T. repens, C. leptophyllum, S. nigrum, Verbena* sp., *Hydrocotyle* sp., *P. americana*) (Table 3). This may be due to the seasonal variation in the seed bank.

From the present study, when herbicides were used to control mature fireweed plants, the established kikuyu pasture was able to significantly suppress the recruitment of new fireweed plants from the seed bank. However, even without herbicide application (as seen in the control plots; Table 3), a dominant kikuyu pasture can reduce fireweed recruitment and seed input. Perennial pasture species [*viz*. setaria (*Setaria sphacelata* Schum.), kikuyu (*Pennisetum clandestinum* Hochst. ex Chiov.), paspalum (*Paspalum dilatatum* Poir.), and Rhodes grass (*Chloris gayana* Kunth.)], that are competitive through late Summer and Autumn, will help to prevent the establishment of fireweed seedlings in the Autumn and Winter months [22]. Therefore, in a field situation, a well-established pasture community, given time, may well prevent further fireweed seedlings from emerging if stocking rates are sensibly managed [26].

## 4. Materials and Methods

### 4.1. Field Experiments

#### 4.1.1. Study Site

A field site at Beechmont (28°5′32.61′′ S; 153°13′11.14′′ E) in Southeast Queensland (SEQ), containing a dense infestation of fireweed (more than 90% of plants were adult flowering plants), was selected for this study (density of ca. 10 to 18 plants m^−2^). The soil was a well-drained red ferrosol with a clay loam-to-clay texture and was dominated by kikuyu grass (*Pennisetum clandestinum* Hochst. ex Chiov). Several other key species were observed (Table 2). The climate was warm temperate, with rainfall averaging 656.5 mm annually [12]. The mean annual maximum temperature was 22.5 °C while the mean annual minimum temperature was 14.0 °C in the year of study. At the beginning of the experiment, monthly rainfall was 16.4 mm, and minimum and maximum monthly temperatures averaged 3.8 and 21.6 °C, respectively [12].

#### 4.1.2. Experimental Design

The experiment was established in June 2018 using a split-plot design, with herbicide treatments allocated to main plots, several applications allocated to sub-plots and each treatment replicated five times. Herbicide treatments comprised the application of either bromoxynil, fluroxypyr/aminopyralid, metsulfuron-methyl or triclopyr/picloram/aminopyralid, plus an untreated control (without herbicide). All herbicides were applied at their recommended rates for the management of fireweed (Table 5). Sub-plot treatments were either a one-off herbicide application (July 2018) or two applications whereby the herbicide treatment was repeated 3 months after the first application (i.e., in October 2018). To set up the trial, five treatment blocks were established parallel to each other, with main plots 10 × 10 m in size and divided into two 5 × 5 m sub-plots. The site was fenced, and livestock was excluded for the full duration of the study (ca. 10 months).

#### 4.1.3. Herbicide Application

The herbicides were applied using a Makita EVH2000 24.5 cm^3^, four-stroke petrol backpack sprayer equipped with a 2.5 m swath hand-held boom containing four nozzles (spaced 50 cm apart) and delivering a carrier spray volume of 800 L ha^−1^ (2.0 L 25 m^−2^ plot). A single pass of the boom was undertaken when spraying plots, with the height maintained at 0.5 m above the soil level by attaching a weighted 50 cm vertical guide to the boom. A pressure gauge mounted on the handle of the boom provided confirmation of an operating pressure of 120 kPa, and a portable metronome ensured a constant walking speed of 4.0 km hour^−1^ was maintained. The Turbo Twinjet flat spray nozzles (TTJ60-11002) used in this experiment were supplied by Spraying Systems (Wheaton, IL, USA). All solutions contained a 2% (*v*/*v*) Pulse Penetrant^®^ (1.0 kg L^−1^ organo-modified polydimethylsiloxane) obtained from Nufarm Australia Limited (Laverton, North Victoria, Australia).

#### 4.1.4. Plant Density

To determine the effect of the herbicides on fireweed density over time, two quadrats (1.0 × 1.0 m) were placed randomly within each sub-plot, and the density of fireweed plants was determined at 0, 2, 3, 5, 7, 10, and 13 months after herbicide application. To identify dead plants, the outer epidermal layer of the stem of the fireweed plants was scraped away to reveal the inner tissues of the stem. Live stems had a green cambium layer immediately beneath the epidermal layer and green or white tissue inside, whereas dead tissues appeared a distinct brown colour.

#### 4.1.5. Seed Bank Density

Before applying the herbicides to the trial site, soil seed bank samples were taken from each subplot designated to receive the follow-up treatment, as well as the untreated control plots, in July 2018. To take soil samples, two 1 × 1 m quadrats were randomly placed within each of the designated sub-plots, and five cylindrical soil cores (5 cm in diameter and 10 cm deep) were extracted from each quadrat (one from each of the four corners and one from the centre of the quadrat) using a soil corer. Soil cores were then separated into two different depths, 0 to 2 and 2 to 10 cm deep.

The soil seed bank samples, taken from the same depths and from both quadrats, were pooled into one sample for each sub-plot. The two soil samples from the two quadrats were placed into separate plastic bags, sealed, and stored at ambient temperature for 2 to 3 days. They were then spread thinly over a 2 cm layer of a Gatton media compost (Osmocote 8–9 M, Osmocote 3–4 M, Nutricote 7 M, coated iron, moisture aid, dolomite, and Osmoform) contained within shallow germination trays (20 × 25 × 6 cm; w/l/d). Then, all trays were distributed randomly to the top of a greenhouse bench at the University of Queensland, Gatton, in July 2018. The temperature in the greenhouse was maintained close to the outside ambient temperature (mean annual maximum of 26.9 °C, with a mean annual minimum of 13.0 °C). Two control trays were placed among the experimental trays to check for compost or greenhouse seed contaminants. All trays were watered daily to maintain soil moisture at or close to the field capacity. The trays were observed regularly for newly emerging seedlings, and when observed, seedlings were marked with a cocktail stick and initially recorded as either being ‘fireweed’ or ‘other species. Once seedlings were large enough to be identified, then, they were counted and removed. If they could not be identified, representative individuals were planted into pots and grown to maturity for further taxonomic identification using the appropriate literature [27,28]. When seedling emergence ceased, the soil in the trays was dried for 2 weeks before being stirred, rewetted, and inspected for any further seedling emergence over a further 9 months [11].

Soil sampling at the field site was undertaken again in March 2019, 5 months after the follow-up herbicide treatments had been applied in October 2018. The same procedure as described above was used to collect soil samples (from follow-up subplots) and to monitor for seedling emergence in the greenhouse. At this time, the temperature in the greenhouse was maintained close to the outside ambient temperature (the mean maximum during March 2019 was 30.0 °C, with a mean minimum during March 2019 of 17.5 °C).

The species diversity of the soil seed bank was calculated using the Shannon–Weiner Index:

H’= −⅀^s^_i−1_ P_i_ log_e_ P_i_

where S is the number of species and Pi is the proportion of the total of all species’ individuals per quadrat represented by the *i*th species [10].

### 4.2. Statistical Analysis

Plant density data were subjected to analysis of variance (ANOVA) to compare the plant density of different treatments. Means comparisons were through the use of the least significant difference (LSD) test (*p* = 0.05).

An ANOVA was also performed to compare the fireweed seed densities between the two seed banks (before and after spraying) once the data had been transformed to a logarithmic scale. Comparison of treatment means was conducted by the LSD (*p* = 0.05) test. All the data were analyzed using the R statistical software (Version 3.6.3).

## 5. Conclusions

Herbicides fluroxypyr/aminopyralid, bromoxynil, metsulfuron-methyl and triclopyr/picloram/aminopyralid are effective in controlling fireweed plants. Fireweed seeds dominated both the upper (0 to 2 cm) and lower (2 to 10 cm) soil layers, with the highest density of fireweed seeds observed in the upper layer. Even with a high seed load being produced at the Beechmont site, several of the herbicides tested were effective in controlling plants and reducing the density of fireweed seeds entering the soil seed bank. According to the Shannon—Weiner index, except for bromoxynil, all other herbicides did not reduce the biodiversity of the pasture seed community. However, the implementation of a follow-up application of bromoxynil was more effective than the single application. For other tested herbicides, even one application was sufficient to control fireweed plants at all stages of development at the Beechmont site, but this was when a healthy pasture stand was present, and the stocking rate was zero. Thus, for the effective management of fireweed, the application of a single dose of one of three different herbicides (fluroxypyr/aminopyralid, metsulfuron-methyl and triclopyr/picloram/ aminopyralid) when simultaneously ungrazed for a minimum of 10 months can be efficacious. However, given the presence of fireweed in the soil seed bank, subsequent follow-up herbicide control may still be necessary at some stage, depending on prevailing environmental conditions.

Further research is now needed to evaluate the effect of pasture competition on fireweed establishment and to determine pasture density thresholds that can prevent recruitment. Further research is also needed to evaluate the impact of increased grazing pressure, the effect of different kinds of grazing stock (cattle, sheep, or goats) on established populations of fireweed, and how bare and disturbed ground can affect the establishment of fireweed. In addition, besides selecting the most effective herbicide, cost considerations will also need to be included in the decision-making process.

## Figures and Tables

**Table 1:** Germinable soil seed bank species seed density of a fireweed-dominated kikuyu pasture at Beechmont, Queensland, before the application of metsulfuron-methyl, fluroxypyr/aminopyralid, triclopyr/picloram/aminopyralid or bromoxynil, compared with a non-treated control, at two soil depths: upper (0 to 2 cm deep) and lower (2 to 10 cm deep).

		Herbicide Treatment									
Family and Species	Life Form	Metsulfuron- Methyl		Fluroxypyr Aminopyralid		Triclopyr Picloram Aminopyralid		Bromoxynil		Control	
		Upper	Lower	Upper	Lower	Upper	Lower	Upper	Lower	Upper	Lower
		––––––––––––––––––––––––––––––––––––––seeds m^−2^ ––––––––––––––––––––––––									
*** Asteraceae ***											
* Senecio madagascariensis	P/F, W	7710	3769	8740	3494	8128	3535	10,736	3973	8709	3198
* Conyza bonariensis	A/F, W	55	10	132	102	143	81	224	112	81	51
*Cirsium vulgare*	P/F, W	20	20	132	31	81	0	61	10	71	41
*** Fabaceae ***											
*Trifolium repens*	P/F	632	530	407	591	509	580	397	530	519	356
*** Oxalidaceae ***											
* Oxalis dillenii	P/F, W	61	10	31	31	10	41	20	173	31	10
*** Poaceae ***											
* Pennisetum clandestinum	P/G, W	1701	2007	2333	1314	2771	4034	3239	2659	3035	2027
*** Apiaceae ***											
*Cyclospermum leptophyllum*	A/F	0	20	0	10	10	10	10	234	10	0
*** Solanaceae ***											
* Solanum nigrum	PA/S, W	0	0	10	0	0	0	0	0	0	0
*** Verbeaceae ***											
*Verbena* sp.	P/F	0	0	0	0	0	0	0	0	10	51
*** Gentianaceae ***											
* Centaurium erythraea	A/F, W	784	1345	550	540	1151	1579	1385	1436	784	937
*** Araliaceae ***											
*Hydrocotyle* sp.	P, W	20	81	0	10	0	31	0	0	10	0
*** Phytolaccaceae ***											
* Phytolacca americana L.	P/F	0	20	0	0	41	61	0	10	0	31
*** Cyperaceae ***											
* Cyperus rotundus L.	P/F	0	183	20	51	122	346	387	51	71	244
*** Juncaceae ***											
*Juncus* sp.	P/G	0	0	0	0	0	0	10	10	0	0
** Unknown sp. **		10	0	31	31	0	31	31	0	0	20
** Total **		11,193	7995	12,386	6205	12,966	10,329	16,500	9198	13,331	6966
** Grand total **		19,188		18,591		23,295		26,698		23,297	

Life form details for longevity (A = annual or biennial, P = perennial) and for life form (F = forb, G = graminoid, S = shrub) Weed status (W = weed) and * Introduced species.

**Table 2:** Shannon—Wiener index for different treatments metsulfuron-methyl (A), fluroxypyr/ aminopyralid (B), triclopyr/picloram/ aminopyralid (C), bromoxynil (D), and Control (E).

Seed Bank	Layer	Treatment	Shannon Wiener Index
Before spray	Top	A	1.035048
		B	0.933172
		C	1.119198
		D	−0.47028
		E	1.025117
	Bottom	A	1.375892
		B	1.295681
		C	1.428605
		D	−0.299021
		E	1.408365
After spray	Top	A	1.415585
		B	1.647513
		C	1.176353
		D	0.852067
		E	1.281637
	Bottom	A	1.165063
		B	0.967562
		C	1.17289
		D	1.027754
		E	−0.59443

**Table 3:** Species density and change in seed density of germinable soil seed bank species found in a kikuyu pasture at Beechmont, Queensland, after spraying twice with herbicides metsulfuron-methyl, fluroxypyr/aminopyralid, triclopyr/picloram/aminopyralid, bromoxynil, and non-treated control, at two soil depths: upper (0 to 2 cm deep) and lower (2 to 10 cm deep).

Family and Species	Life Form	Metsulfuron-Methyl		Fluroxypyr Aminopyralid		Triclopyr Picloram Aminopyralid		Bromoxynil		Control	
		Upper	Lower	Upper	Lower	Upper	Lower	Upper	Lower	Upper	Lower
		––––––––––––––––––––––––––––––––––––––seeds m^−2^–––––––––––––––––––––––––––––––									
*** Asteraceae ***											
* Senecio madagascariensis	P/F, W	1059 (−39%)	591 (−25%)	601 (−43%)	519 (−35%)	856 (−34%)	805 (−11%)	1070 (−46%)	866 (−26%)	1263 (−28%)	662 (−28%)
* Cirsium vulgare	P/F, W	20 (0)	20 (+1%)	20 (0)	0 (0)	0 (−1%)	10 (0)	0 (0)	10 (0)	20 (0)	(−1%)
* Conyza bonariensis	A/F, W	112 (+1%)	31 (+1%)	20 (0)	0 (−2%)	0 (−1%)	10 (0)	0 (−1%)	10 (−1%)	20 (0)	0 (−1%)
*** Poaceae ***											
* Pennisetum clandestinum	P/G, W	1599 (+29%)	1711 (+40%)	1049 (+30%)	1538 (+43%)	1569 (+32%)	2149 (+23%)	4105 (+54%)	3494 (+42%)	1742 (+28%)	2720 (+46%)
*** Oxalidaceae ***											
* Oxalis dillenii	P/F, W	31 (0)	10 (0)	41 (2%)	10 (0)	10 (0)	51 (+1%)	71 (+1%)	112 (0)	41 (+1%)	61 (+2%)
*** Portulacaceae ***											
* Portulaca oleracea	A/F, W	20	20	10	20	10	20	0	20	10	31
*** Fabaceae ***											
* Medicago polymorpha	A/F, W	744	224	316	265	387	377	183	367	326	112
*** Malvaceae ***											
* Modiola caroliniana	P/F	10	20	31	0	112	20	0	20	0	51
*** Juncaceae ***											
*Juncus sp.*	P/G	0 (0)	10 (0)	10 (0)	0 (0)	0 (0)	20 (0)	0 (+1%)	20 (0)	0 (0)	0 (0)
*** Cyperaceae ***											
*Cyperus rotundus.*	P/F	173 (+5%)	112 (+2%)	411 (+19%)	10 (0)	10 (−1%)	112 (0)	194 (+1%)	132 (+2%)	306 (+8%)	81 (−1%)
*** Gentanaceae ***											
* *Centaurium erythraea*	A/F, W	0 (−7%)	(−17%)	(−4%)	(−9%)	51 (−9%)	(−15%)	(−7%)	102 (−16%)	(−6%)	(−11%)
Total		3595	2637	2149	2413	2964	3462	5562	4929	3422	3637
Grand total		6232		4562		6426		10,491		7059	

Life form entails longevity (A= annual or biennial, P = perennial) and lifeform (F = forb, G = graminoid, S = shrub), Weed status (W = weed) and * introduced species. The percentage reduction (−) and increase (+) of species compared to before spray is shown in parentheses.

**Table 4:** Germinable seed density of fireweed from a kikuyu pasture at Beechmont, Queensland, before and after herbicide treatments compared with total seed density, the percent of total seed density, and the percent reduction of fireweed seed number after herbicide treatment, and reduction in seed density as compared with non-treated control plot. The measurements were taken before and after herbicide treatment and then determined for two soil layers (0 to 2 cm depth and 2 to 10 cm).

Treatment	Timing	Soil Layer	All Species	–––––Fireweed–––––––			
			––––––Seed m^−2^––		Total (%)	Reduction (%)	Control (%)
metsulfuron-methyl	Before	Upper	11,193	7710 ^a^	69	−40	
	After		3595	1059 ^cde^	29		** −12 **
	Before	Lower	7995	3769 ^b^	47	−25	
	After		2637	591 ^efg^	22		+3
fluroxypyr/aminopyralid	Before	Upper	12,386	8740 ^a^	71	−43	
	After		2149	601 ^fg^	28		** −15 **
	Before	Lower	6205	3494 ^b^	56	−34	
	After		2413	519 ^g^	22		** −6 **
triclopyr/picloram/aminopyralid	Before	Upper	12,966	8128 ^a^	63	−34	
	After		2964	856 ^cdef^	29		** −6 **
	Before	Lower	10,329	3535 ^b^	34	−11	
	After		3462	805 ^cdefg^	23		+17
bromoxynil	Before	Upper	16,500	10,736 ^a^	65	−46	
	After		5562	1070 ^d^	19		** −18 **
	Before	Lower	9198	3973 ^b^	43	−25	
	After		4929	866 ^cdef^	18		** +3 **
Control	Before	Upper	13,331	8709 ^a^	65	−28	
	After		3422	1263 ^c^	37		
	Before	Lower	6966	3198 ^b^	46	−28	
	After		3637	662 ^defg^	18		

Seed densities in each row, followed by different letters, are significantly different (*p* < 0.05).

**Table 5:** Herbicide treatments, trade names, and rates applied to control fireweed. All treatments included surfactant; 2% (*v*/*v*) Pulse Penetrant^®^ (1 kg L^−1^) organo-modified poly dimethyl siloxane, Nufarm Australia Limited, Laverton, North Victoria, Australia.

Herbicide	Trade Name	Rate	Product Rate
		(g Active Ingredient ha^−1^)	(25 m^−2^)
bromoxynil	Bromicide^®^ 200, Nufarm Australia Limited, Victoria, Australia	560	7.00 mL
metsulfuron-methyl	Brush-Off^®^, Bayer, Victoria, Australia	24	0.10 g
fluroxypyr/aminopyralid	HotShot™, CortevaTM Agriscience, New South Wales, Aus-tralia	210/15	3.75 mL
triclopyr/picloram/aminopyralid	Grazon™ Extra, Cor-tevaTM Agriscience, New South Wales, Australia	900/300/24	7.50 mL
